# The Causal Effect of Student Mobility on Standardized Test Performance: A Case Study with Possible Implications for Accountability Mandates within the Elementary and Secondary Education Act

**DOI:** 10.3389/fpsyg.2016.01096

**Published:** 2016-07-19

**Authors:** Arielle S. Selya, Eden Engel-Rebitzer, Lisa Dierker, Eric Stephen, Jennifer Rose, Donna L. Coffman, Mindy Otis

**Affiliations:** ^1^Department of Population Health, University of North DakotaGrand Forks, ND, USA; ^2^Department of Psychology, Wesleyan UniversityMiddletown, CT, USA; ^3^Department of Epidemiology and Biostatistics, College of Public Health, Temple UniversityPhiladelphia, PA, USA; ^4^Department of Pupil Services and Special Education, Middletown Public SchoolsMiddletown, CT, USA

**Keywords:** academic performance, elementary and secondary education act, every student succeeds act, high school, no child left behind, propensity score methods, standardized tests, student mobility

## Abstract

This paper presents a limited case study examining the causal inference of student mobility on standardized test performance, within one middle-class high school in suburban Connecticut. Administrative data were used from a district public high school enrolling 319 10th graders in 2010. Propensity score methods were used to estimate the causal effect of student mobility on Math, Science, Reading, and Writing portions of the Connecticut Academic Performance Test (CAPT), after matching mobile vs. stable students on gender, race/ethnicity, eligibility for free/reduced lunches, and special education status. Analyses showed that mobility was associated with lower performance in the CAPT Writing exam. Follow-up analyses revealed that this trend was only significant among those who were ineligible for free/reduced lunches, but not among eligible students. Additionally, mobile students who were ineligible for free/reduced lunches had lower performance in the CAPT Science exam according to some analyses. Large numbers of students transferring into a school district may adversely affect standardized test performance. This is especially relevant for policies that affect student mobility in schools, given the accountability measures in the No Child Left Behind that are currently being re-considered in the recent Every Student Succeeds Act.

## Introduction

The US public school system is struggling in many areas, with growing achievement gaps and falling international rankings. While a wide range of factors contribute to schools' success and students' well-being, one important factor is student mobility across schools or school districts. Many studies have shown that student mobility correlates with a variety of negative outcomes, including an increased risk of psychological and behavior problems (Simpson and Fowler, 1994; Ellickson and McGuigan, 2000; Rumberger, 2003) and disciplinary action (Engec, 2006), as well as adverse educational outcomes such as dropping out of high school (Rumberger and Larson, 1998) and lower test performance (Mehana and Reynolds, 2004; Engec, 2006; Strand and Demie, 2007; Thompson et al., 2011; Voight et al., 2012).

However, the fact that mobile and stable students are typically two very different populations presents a serious problem for measuring the true effect of mobility on educational performance. Because mobile students are more likely to be in a lower socioeconomic status and have poorer academic performance to begin with (Nelson et al., 1996; Temple and Reynolds, 1999), it remains unclear whether mobility has an effect on student performance above and beyond these preexisting differences. Fortunately, innovative statistical methods have the potential to more accurately estimate the causal effects of student mobility on academic performance, provided all confounding variables are measured.

The question of whether student mobility has a causal effect on academic performance is especially interesting in the context of the Elementary and Secondary Education Act (ESEA). The ESEA is an overarching bipartisan measure originally passed in 1965, which was re-authorized in the recent forms of the No Child Left Behind (NCLB) Act from 2002, and more the Every Student Succeeds Act (ESSA) in 2015. One of NCLB's central features was increased accountability of schools in tracking student achievement. This law measured achievement by a set of statewide exams administered annually between 3rd and 8th grade and at least once during high school. Beginning in 2002, schools were given 12 years to reach 100% proficiency on these exams, as determined by state set proficiency goals (Kim and Sunderman, 2005). Each school was also responsible for making adequate yearly progress (AYP) toward the goal of 100% proficiency. Schools that met state goals received bonuses and federal recognition. On the other hand, failure to meet AYP introduced penalties for the school, which can vary in severity by state. In Connecticut under NCLB, schools that failed to meet goals for 2 consecutive years were labeled “in need of improvement” and were required to develop an improvement plan and allow any interested students to move to a higher performing school. If a school did not meet AYP for a third year, it was required to provide free tutoring, and after a fourth year the school was potentially subject to staff replacement, a new curriculum, or extension of classroom time. A restructure was to be planned and implemented if the school did not meet this goal for a fifth and sixth year.

NCLB was a highly controversial policy, especially with respect to these accountability mandates. Some evidence shows that NCLB produced increases for some, notably in math achievement, especially among low-achieving groups (Dee and Jacob, 2011). On the other hand, critics of NCLB argue that AYP requirements have unequal effects on schools: for example, schools with lower initial achievement levels (Brown and Clift, 2010) and schools in rural settings (Zhang and Cowen, 2009) suffer disproportionately. Critics also argue that these requirements may have introduced unanticipated side effects on education. For example, teachers may respond to this pressure by “teaching to the test” (Jones et al., 2003; Desimone, 2013), that is, aligning instruction to the test's expected standards and format, which can compromise deeper or more holistic thinking and skills in students (MacPherson, 2009; Liu and Neilson, 2011; Jensen et al., 2014) as well as the validity of standardized testing (Downing, 2002; Jennings and Bearak, 2014). Moreover, since NCLB allows states to develop their own testing standards to measure student performance and AYP, there is a concern that states could react by lowering their standards (Ryan, 2004). In fact, the state of Connecticut cited concerns that certain changes suggested in order to achieve compliance with NCLB would lower testing standards in a lawsuit filed against the federal government over how to fund the additional testing required (Connecticut vs. Spellings, 2005).

Since the passage of NCLB, several events have impacted the accountability mandates and AYP requirements. In 2012, President Obama granted waivers from NCLB requirements to many states, including Connecticut, which in exchange implemented the Common Core standards (U.S. Department of Education, 2012). More recently, the recent passage of ESSA in 2015 seeks to mitigate some of NCLB's detrimental outcomes by providing states with more flexibility about how to identify and support the most struggling schools. As a result, states are currently re-evaluating the requirements and consequences of student performance and AYP as they plan and implement ESSA.

An additional concern for states to consider during planning and implementation of new accountability requirements under ESSA is that AYP as originally specified in NCLB is measured at the level of the school rather than at the level of the child. That is, AYP compares the scores of one grade level of students across years. This measure of progress is potentially problematic: if the population of students changes from year to year, AYP is not a valid assessment of the school's progress in moving individual students toward proficiency. In addition, when a school invests resources in a group of students, only the gains of the students still in the district during the state exam will be reflected at the time of testing. Conversely, students transferring into the district will affect a school's performance, especially if mobility causes or is associated with lower academic performance.

Taken together, NCLB's original accountability mandates have serious consequences for schools which states should consider when implementing new mandates for ESSA. In particular, if student mobility negatively impacts performance on statewide exams, this will most likely contribute to unfavorable AYP performance. Under NCLB requirements, this would subject the school to penalties, with the problem being worst for schools faced with high levels of student mobility. For example, as a result of the 1996 *Sheff v. O'Neill* case in which the Connecticut Supreme Court mandated redistricting to ensure a more balanced racial/ethnic distribution of students within schools, substantial student transfer between public schools in Connecticut has taken place, particularly of minority children. Unfortunately, there may be unforeseen negative consequences related to these high levels of student mobility.

Several studies have attempted to isolate the true effect of mobility by controlling for demographic and socioeconomic variables in their analyses. Many of these studies failed to show that mobile vs. stable students had significantly different academic performance once these variables were controlled for (Alexander et al., 1996; Wright, 1999; Strand and Demie, 2006), a fact which raises doubts about the true effect of mobility. Though other studies did report a significant effect of mobility even after controlling for potential confounding variables (Strand and Demie, 2007; Thompson et al., 2011; Grigg, 2012; Herbers et al., 2012; Parke and Kanyongo, 2012), the standard practice of statistically controlling for confounding may fail to eliminate the inherent bias if the two groups are very different from each other with respect to these confounding variables. Propensity score methods are more appropriate in such cases for estimating causal effects: they essentially re-sample the dataset so that “treatment” (here, mobility) and “control” (here, stable) groups are equivalent with respect to all measured confounding variables, thus approximating the results of a randomized study design, and allowing causal inferences to be drawn, provided there are no unmeasured confounding variables (Rosenbaum, 2002, 2010). Though propensity score methods have been used to examine the effect of mobility on high school dropout (Gasper et al., 2012), very little is currently known about the true causal effect of mobility on standardized test performance, given that the majority of studies on student mobility have used conventional statistical techniques which most likely yield biased effect estimates.

This study uses administrative data from a middle-class public high school in Connecticut as a limited case study that uses causal inference to estimate the effects of mobility on students' academic performance. Propensity score methods are utilized to evaluate whether transferring into a school district during middle or high school has a causal effect on numerical scores and rates of proficiency on the state mandated standardized exams in 10th grade. Both (1) 1-nearest neighbor propensity score matching and (2) “full matching” of stable to mobile students were performed in order to match mobile and stable groups in terms of gender, race/ethnicity, eligibility for free/reduced lunch, and special education status. We hypothesized that propensity score methods would show a detrimental causal effect of mobility on standardized test performance, which would in turn show that schools facing high levels of student mobility may be more adversely affected by the accountability mandates of NCLB and potentially ESSA.

## Materials and methods

### Sample

The sample included all enrolled 10th grade students from a district public high school in Central Connecticut who were administered the State's 10th grade standardized achievement test for the first time in 2010 (*N* = 319). A total of 48.8% were female. In terms of ethnicity, 54.9% were Caucasian, 10.9% Hispanic, 28.5% African American, 5.5% Asian, and 0.3% American Indian. Due to small sample sizes, Asian and American Indian students were removed from all analyses, for a final sample size of *N* = 302. In addition, 36.7% were eligible for free/reduced school lunches and 13.9% were receiving special education. In 2010–2011, the high school represented by the present data was in its sixth year of failing to make AYP, despite an overall increased performance across Connecticut from 60 to 72% of schools meeting AYP standards over the prior 2 years.

### Measures

#### Academic performance

The State of Connecticut complies with NCLB by administering standardized tests in the spring of each academic year to students in grades three through eight (Connecticut Mastery Test-CMT) and grade 10 (Connecticut Academic Performance Test-CAPT). The CAPT measures *Math achievement* in four content areas (algebraic reasoning, numerical and proportional reasoning, geometry and measurement, and working with data) through multiple choice and open-ended questions. *Science achievement* is measured across five content areas (energy transformations, chemical structures and properties, global interdependence, cell chemistry and biotechnology, and genetics evolution, and biodiversity) through multiple choice and open-ended questions. *Reading achievement* is measured across two content areas (response to literature and reading for information) through a combination of multiple choice, short answer, and essay questions. *Writing achievement* is measured across two content strands (interdisciplinary writing and editing and revising) through essays and multiple-choice questions.

Each area of achievement is scored out of a total of 400 points and categorized into five levels (below basic, basic, proficient, goal, and advanced), reflecting State-established proficiency guidelines.

Fully 93% *(N* = 297) of 10th graders completed the standardized Math section (*M* = 239.9, *SD* = 41.0, range = 145–347), with 66.7% achieving proficient or above; 97% (*N* = 311) completed the Science section (*M* = 249.7, *SD* = 47.8, range = 100–391), with 75.6% achieving proficient or above; 93% (*N* = 297) completed the Reading section (*M* = 230.1, *SD* = 42.6, range = 129–353) with 70.7% achieving proficient or above; and 94% (*N* = 301) completed the Writing section (*M* = 248.7, *SD* = 56.3, range = 100–400) with 74.4% achieving proficient or above. Ninety percent (*N* = 289) of 10th graders completed the entire standardized state exam with 54.2% (*N* = 173) reaching proficient or above in each of the four areas.

#### Mobility

Students were categorized as *stable* if they were present in the school district from grade 6 through grade 10 and *mobile* if they transferred into the district during middle school (grade 7 or 8) or high school (grades 9 or 10). Sixty-four percent (*N* = 204) of students were classified as stable and the remaining 36% as mobile (*N* = 115) with 17.4% (*N* = 20) of mobile students entering the district during middle school, 59.1% (*N* = 68) entering during high school and another 23.5% (*N* = 27) having left and reentered the district between 6th and 10th grade. When first tested within the district, mobile students showed lower rates of proficiency than did stable students in grade 6 (Math, 64% of mobile vs. 78% of stable students were proficient, $X_{\left(1\right)}^{2}$ = 6.20, *p* = 0.01 and Reading, 59% of mobile vs. 71% of stable students were proficient, $X_{\left(1\right)}^{2}$ = 2.37, *p* = 0.12). While the effect for the content area of Reading was in the expected direction, the effect did not achieve statistical significance.

#### Student characteristics used in propensity score matching

Student's eligibility for free or reduced lunch was coded dichotomously as eligible (yearly income of less than $7090 plus $3740 for every person living in the house) or ineligible (yearly income above that threshold; U.S. Department of Agriculture, 2011). Special education status was a dichotomous variable indicating whether or not the student was enrolled in special education. Ethnicity was coded dichotomously for White, African-American, and Hispanic. Because of the very small sample of Asian Americans and Native Americans, these participants were excluded from all analyses.

#### Analyses

The association between mobility and student achievement (i.e., Math, Science, Reading, and Writing) was first modeled with free/reduced lunch status, gender, ethnicity, special education status, the interaction between mobility and free/reduced lunch eligibility, and the interaction between mobility and special education status included as covariates. Multiple regression was used to investigate the numeric score of tests within each subject area, and logistic regression was used to investigate rates of proficiency in each subject area.

Next, propensity score methods for causal inference were used in order to estimate the causal effect of mobility on CAPT performance. Propensity score methods essentially re-sample the data such that they approximate the dataset, had random assignment to “treatment” (i.e., mobility) and “control” (i.e., staying in the same school) conditions been possible; as a result, they allow the true causal effect to be estimated more accurately than simply controlling for potential confounding variables (Rosenbaum, 2002, 2010). Since there are several different types of propensity score methods, with some disagreement as to the optimal method, two different types are presented here in order to evaluate the robustness of the results across different types causal inference methods.

First, nearest-neighbor matching of propensity scores was performed. Propensity scores quantify each students' likelihood of mobility based on other student characteristics that are potential confounding variables of both test performance and mobility. Available variables included gender (females tend to have higher performance on tests), ethnicity (a result of the *Sheff v. O'Neill* ruling in which minorities are disproportionately more likely to be transferred to new schools), free/reduced meal eligibility (an indicator of socioeconomic status), special education status (which may prompt school transfers by teachers, administrators, and/or parents), the interaction between free/reduced meal eligibility and ethnicity (since socioeconomic status may impact whites' and minorities' likelihood of mobility differently), and the interaction between free/reduced meal eligibility and special education (since students in special education may have different likelihoods of mobility depending on socioeconomic status). Propensity scores were calculated as the fitted values of a logistic regression of mobility on all of these terms, and were calculated separately for each CAPT subject area, since occasional missing data resulted in slightly different sets of observations for each. Stable students were matched to mobile students based on propensity scores, using the Matching package in R (Sekhon, 2011) with 1-nearest neighbor matching, without replacement, constrained to a caliper of 0.1 standard deviations of the propensity score. This process seeks to create two groups that are equivalent on all characteristics except treatment (i.e., mobility), allowing a causal effect on CAPT performance to be estimated, provided there are no unmeasured confounding variables. To ensure this equivalency, the matched groups were evaluated to check whether balance was achieved successfully; that is, whether there were no remaining significant differences in covariates between stable and mobile students. Finally, the average treatment effect on the treated (ATT), i.e., the estimated causal effect of mobility, was estimated once successful matching was achieved.

Second, the method of “full matching,” which forms many small subclasses in an optimal way (Rosenbaum, 2002; Hansen, 2004), was used to create a matched dataset in which one or more stable students are matched to each mobile student based on all of the variables described above which were used to calculate propensity scores. The process of full matching produces frequency weights, which weight the dataset such that it approximates the results of random assignment to treatment and control conditions. Full matching was implemented using the R package MatchIt (Ho et al., 2011), for each CAPT subject area. The ATT of mobility was subsequently estimated using weighted regression models of test performance on mobility, in which the frequency weights from full matching were used. Multiple regressions were used for numeric test scores, and logistic regressions were used for proficiency rates.

## Results

Table 1 presents individual student characteristics by mobility status. Mobile students had higher proportions of males, African-Americans, and free/reduced lunch eligibility, compared to the proportions among stable students. Stable students had higher proportions of White students than did mobile students.

**Table 1:** **Student characteristics by mobility status**.

	**Mobile students (*N* = 109)**	**Stable students (*N* = 193)**	**Chi-Square statistics**
	***N* (%)**	***N* (%)**	***X*^2^(d.f.), *p***
Gender (% Male)	60 (55.05)	93 (48.19)	1.31(1), 0.25
**ETHNICITY**			
White	55 (50.46)	120 (62.18)	3.92 (1), 0.05^*^
Black	41 (37.61)	50 (25.91)	4.54 (1), 0.03^*^
Hispanic	13 (11.93)	23 (11.91)	<0.0001 (1), 0.99
Special Education	21 (19.27)	23 (11.92)	3.02 (1), 0.08
Free/reduced lunch eligible	50 (45.87)	61 (31.61)	6.10 (1), 0.01^*^

* *p < 0.05*.

Multiple and logistic regression analyses examined the association between student mobility and test performance in all four subject areas of the CAPT, after simultaneously controlling for gender, ethnicity, free/reduced lunch eligibility and special education, and interactions between mobility and each of free/reduced lunch eligibility and special education status. Results (Table 2) revealed that mobile students scored lower on Writing (*B* = −11.85, *p* = 0.04), and were less likely to reach proficiency in Writing (*OR* = 0.49, *CI* = [0.26–0.91], *p* = 0.03). Lower scores on Writing were also predicted by Black (*B* = −32.86, *p* < 0.01) and Hispanic (*B* = −26.91, *p* = 0.01) ethnicity relative to White ethnicity, placement in special education (*B* = −46.14, *p* < 0.01), and eligibility for free/reduced meals (*B* = −22.11, *p* = 0.01), while being female was associated with higher scores on Writing (*B* = 27.10, *p* < 0.01). Lower rates of Writing proficiency were also predicted by Black (vs. White) ethnicity (*OR* = 0.36, *CI* = [0.17–0.77], *p* = 0.01) and placement in special education (*OR* = 0.11 *CI* = [0.04–0.26], *p* < 0.01), while being female was associated with higher rates of Writing proficiency (*OR* = 3.11, *CI* = [1.65–5.87], *p* < 0.01).

**Table 2:** **Results of multiple and logistic regressions examining the relationship between student mobility and achievement scores**.

	**Multiple regression of numeric scores**				**Logistic regression of proficiency rates**			
	**Math score**	**Science score**	**Reading score**	**Writing score**	**Math prof**.	**Science prof**.	**Reading prof**.	**Writing prof**.
Mobility	−6.75	−7.15	−5.79	−11.85^*^	0.81	0.61	0.70	0.49^*^
Female gender	−0.84	0.35	20.22^***^	27.10^***^	1.12	0.79	3.13^***^	3.11^***^
Black ethnicity^†^	−29.37^***^	−33.64^***^	−23.82^***^	−32.86^***^	0.22^***^	0.22^***^	0.42^*^	0.36^**^
Hispanic ethnicity^†^	−21.59^**^	−29.54^***^	−24.41^**^	−26.91^**^	0.22^**^	0.31^*^	0.26^**^	0.79
Free/reduced meals	−17.05^***^	−18.11^**^	−17.05^**^	−22.11^**^	0.47^*^	0.52	0.50^*^	0.54
Special education	−36.26^***^	−41.27^***^	−32.46^***^	−46.14^***^	0.12^***^	0.10^***^	0.25^**^	0.11^***^

*Left 4 columns: regression coefficients from multiple regression of numeric achievement scores. Right 4 columns: odds ratios from logistic regression of rates of proficiency. Rows show all covariates*.

† vs. White ethnicity;

* p < 0.05;

** p < 0.01;

*** *p < 0.001*.

Next, propensity scores were calculated to quantify each student's propensity for being mobile. In all CAPT subject areas, 1-nearest neighbor matching was successful in eliminating all prior significant differences among predictors of mobility. That is, after matching, mobile and stable students did not differ in terms of gender, ethnicity, special education, free/reduced lunch eligibility, the interaction between ethnicity and free/reduced lunch eligibility, or the interaction between special education and free/reduced lunch eligibility (all *p* > 0.05 and standardized mean differences <0.1). After matching students on these propensity scores, average treatment effect on the treated (*ATT*) of mobility was estimated in terms of absolute performance differences (Table 3). Mobility was significantly associated with poorer performance on Writing, as measured by both lower average scores (*ATT* = −15.39, standard error (*SE*) = 6.44, *p* = 0.02), and a lower rate of proficiency (odds ratio for *ATT* (*OR*) = 0.88, 95% confidence interval (*CI*) = [0.78–1.00], *p* = 0.04). That is, mobile students scored on average 15.4 points lower on the Writing test, and were only 88% as likely to reach Writing proficiency as stable students were. Additionally, mobility showed a trend with poorer performance in Science, such that mobile students scored on average 10.2 points lower (*SE* = 5.42, *p* = 0.06) and were likely to reach proficiency in Science only 89% as often (*OR* = 0.89, *CI* = [0.80–1.01], *p* = 0.07) as stable students. Mobility was not significantly associated with performance differences in Math or Reading after matching on propensity scores.

**Table 3:** **ATT of student mobility on test scores after 1-nearest neighbor matching on propensity scores**.

	**Linear regression of numeric scores**				**Logistic regression of proficiency**			
	**Math Score**	**Science Score**	**Reading Score**	**Writing Score**	**Math Prof**.	**Science Prof**.	**Reading Prof**.	**Writing Prof**.
ATT	−5.12	−10.19	−8.14	−**15.39**	0.99	0.90	0.95	**0.88**
95% CI	−15.52 to 5.01	−20.82 to 0.42	−18.96 to 2.76	−**27.94** to −**2.86**	0.86 to 1.13	0.80 to 1.01	0.84 to 1.07	**0.78** to **1.00**
*p*-value	0.32	0.06	0.14	0.02^*^	0.62	0.07	0.38	0.04^*^

*Average treatment effect on the treated (ATT) was obtained from students' numeric scores (left 4 columns) and binary indicators of achieving proficiency (right 4 columns) after matching students on propensity scores for student mobility. ATT shows the decline in numeric scores (left 4 columns; based on linear regression) and the odds ratio of attaining proficiency (right 4 columns; based on logistic regression) associated with mobility. Ninety-five percent confidence intervals (CI) and p-values are shown for numeric scores and rates of proficiency in 4 academic subject areas. Bold*,

* *p < 0.05*.

Finally, full matching was used to create a matched dataset in which each mobile student was matched with one or more stable students on the combination of factors that were previously used to calculate propensity scores. Matching successfully eliminated differences on covariates between mobile and stable students in the full dataset (all standardized mean differences after matching <0.05). Based on weighted regressions using the frequency weights from full matching (Table 4), the estimated ATT of mobility on test performance revealed that student mobility was associated with lower scores in Writing scores by 13.4 points (*SE* = 6.56, *p* = 0.04). Additionally, mobile students trended toward having lower rates of proficiency in Science (*OR* = 0.55, *CI* = [0.28–1.09], *p* = 0.08) and Writing (*OR* = 0.56, *CI* = [0.30–1.06], *p* = 0.07).

**Table 4:** **ATT of student mobility on test scores based on a matched dataset created by full matching**.

	**Multiple regression of numeric scores**				**Logistic regression of proficiency**			
	**Math Score**	**Science Score**	**Reading Score**	**Writing Score**	**Math Prof**.	**Science Prof**.	**Reading Prof**.	**Writing Prof**.
ATT	−5.66	−7.82	−1.21	−**13.46**	0.93	0.55	0.92	0.56
95% CI	−15.56 to 4.24	−18.57 to 2.93	−11.48 to 9.05	−**26.33** to −**0.60**	0.51 to 1.73	0.28 to 1.09	0.47 to 1.78	0.30 to 1.06
*p*-value	0.26	0.16	0.82	0.04^*^	0.83	0.08	0.80	0.07

*Matched dataset matched stable students to mobile students on gender, ethnicity, free/reduced lunch eligibility, and special education status. Mean average treatment effect on the treated (ATT) of mobility was obtained from the coefficients from a linear regression model of numeric test scores on mobility (left 4 columns) and odds ratios from a logistic regression model of the rate of proficiency on mobility (right 4 columns). Confidence intervals (CI) and p-values are also shown. Bold*,

* *p < 0.05*.

Follow-up analyses were performed on separate groups based on their eligibility for free/reduced lunch. Among those who were eligible for free/reduced lunch (i.e., low-income students), mobility was not found to significantly affect performance in Math, Reading, or Writing (all *p* > 0.10; individual results not shown). Though the association with numeric Science scores reached significance, this result is inconclusive because matching was not successfully achieved with the available covariates. On the other hand, mobility significantly impaired some measures of performance for students who were *ineligible* for free/reduced lunch (Table 5). Specifically, both nearest-neighbor and full matching indicated that mobility was associated with lower Writing scores by 24.17 points (*SE* = 9.91, *p* = 0.01) and 26.40 points (*SE* = 9.36, *p* = 0.01), respectively, and showed a trend toward lowering the odds of achieving proficiency in Writing (both *p* < 0.10). Additionally, full matching, but not nearest-neighbor matching, indicated that mobility decreased Science scores by 20.83 points (*SE* = 8.50, *p* = 0.01) as well as the odds of achieving proficiency in Science (*OR* = 0.24, *CI* = [0.09–0.66], *p* = 0.01).

**Table 5:** **ATT of student mobility on test scores among students who were ineligible for free/reduced lunch, according to both nearest-neighbor (NN) matching (top rows) and full (F) matching (bottom rows)**.

	**Multiple regression of numeric scores**				**Logistic regression of proficiency**			
	**Math Score**	**Science Score**	**Reading Score**	**Writing Score**	**Math Prof**.	**Science Prof**.	**Reading Prof**.	**Writing Prof**.
**NN:** ATT	−12.61	−10.17	−12.02	−**24.17**	0.92	0.86	0.94	0.88
95% CI	−29.45 to 4.32	−26.82 to 6.49	−26.52 to 2.48	−**43.60** to −**4.74**	0.79 to 1.08	0.74 to 1.00	0.81 to 1.10	0.76 to 1.02
*p*-value	0.14	0.23	0.10	0.02^*^	0.17	0.05	0.46	0.08
**F:** ATT	−7.42	−**20.83**	−11.08	−**26.40**	0.77	**0.24**	0.714	0.42
95% CI	−21.36 to 6.53	−**35.65** to −**6.00**	−24.74 to 2.57	−**44.70** to −**8.11**	0.31 to 1.94	**0.09** to **0.66**	0.25 to 1.97	0.17 to 1.04
*p*-value	0.30	0.01^*^	0.11	0.01^*^	0.58	0.01^*^	0.51	0.06

*Matched dataset matched stable students to mobile students on gender, ethnicity, and special education status. Mean average treatment effect on the treated (ATT) of mobility was obtained from the coefficients from a linear regression model of numeric test scores on mobility (left 4 columns) and odds ratios from a logistic regression model of the rate of proficiency on mobility (right 4 columns). Confidence intervals (CI) and p-values are also shown. Bold*,

* *p < 0.05*.

## Discussion

This paper examined the relationship between student mobility and academic achievement in grade 10 within a small, middle-class high school in Connecticut. Transferring into the district during middle school or high school was found to be associated with decreased scores and rates of proficiency in 10th grade state standardized tests in Writing, relative to staying in the school district since at least grade 6. These estimated causal effects of mobility held after successfully eliminating differences in demographic and background characteristics within matched samples of mobile and stable students. Follow-up analyses stratified by eligibility for free/reduced lunch showed that mobility was associated with significantly poorer performance in Writing and Science for ineligible, but not eligible (low-income), students. Given these findings that mobility is linked to decreased proficiency rates in some subject areas, mobility is likely to have contributed to this school's lower AYP.

Previous research has yielded mixed results regarding whether mobility has a significant association with or effect on student academic achievement. On one hand, several studies have argued that much of the relationship between mobility and academic achievement can be explained by confounding factors such as student characteristics and socioeconomic status and prior test performance (Dauber et al., 1996; Rumberger and Larson, 1998; Temple and Reynolds, 1999; Wright, 1999; Strand and Demie, 2006). On the other hand, numerous other studies have found a significant effect of student mobility on academic achievement, even after controlling for known confounding variables (Strand and Demie, 2007; Thompson et al., 2011; Gasper et al., 2012; Parke and Kanyongo, 2012). Importantly, one of these studies used propensity score matching to show that, after matching on background and demographic characteristics, mobility increased the likelihood of dropping out of high school (Gasper et al., 2012). Similarly, the current study's use of propensity score methods provides strong evidence that, even after adjusting for demographic factors, mobility is associated with poorer performance on standardized tests. The current study reinforces previous findings that mobility may in part cause lower performance on standardized tests. Given that many previous studies used potentially biased standard regression techniques, the current study's use of propensity score methods provides especially strong evidence for a less-biased estimate of the causal effect of mobility.

Several studies have hypothesized how mobility may negatively impact student performance. For example, social capital theory postulates that changing schools breaks important social ties among and between students, teachers, and parents, increasing the risk of dropping out of high school (Coleman, 1988). In support of this, longitudinal studies found that moving results in decreased school performance due to disrupted social relationships (Pribesh and Downey, 1999; South et al., 2007). Alternatively, it is possible that mobility has a detrimental effect on academic achievement due to the disrupted curricula and/or assessment practices resulting from changing schools. For example, there may be a delay introduced by teachers' need to become familiar with new students' educational performance (Gasper et al., 2012), or the student may miss key concepts that are necessary for future skills (Kerbow et al., 2003). Current knowledge on these mechanisms of mobility's detrimental effect on school performance can be strengthened by future studies using administrative data of students that utilize causal inference methods and mediation models.

The current findings indicate that student mobility most consistently affects performance in Writing. This could be due to a relative neglect of teaching writing skills in high schools across the country, in favor of teaching reading and mathematics (The National Commission on Writing, 2002). The CAPT Writing exam requires skills well beyond basic language skills: students must comprehend two texts presenting two sides of an issue, think critically about them, choose a side, formulate an argument, and write a persuasive essay. Such interdisciplinary writing skills are often neglected in high schools, where among a body of discipline-specific teachers, it is unclear whose responsibility it is to teach writing skills (Alliance for Excellent Education, 2006), and students in recent years are given writing assignments only infrequently, and only short ones at that (Applebee and Langer, 2009). It is therefore possible that since interdisciplinary writing requires an accumulation of various and interdisciplinary skills, mobility is particularly disruptive to performance on the Writing portion of the CAPT.

The result that the academic performance of students *ineligible* for free/reduced lunch was affected by mobility, but not of eligible students, was unexpected considering previous evidence suggesting that low-income students are disproportionately more affected by mobility (Temple and Reynolds, 1999). One possible explanation is that mobility's disruptive effects are apparent only at higher levels of performance; considering higher-income students tend to have better academic performance overall, this would explain why our findings identified a negative effect of mobility only among those students whose income was high enough to disqualify them from receiving free/reduced lunches. Alternatively, however, it is possible that the current sample from a single middle-class school, underestimates the effect of mobility on low-income students and thus cannot be appropriately used to examine this question.

Another notable result among students ineligible for free/reduced lunch was the possible decline in performance on the CAPT Science tests, in addition to the effects on Writing. The explanation that mobility's disruptive effects are only apparent at higher levels of performance is also viable for this result, considering that of the four subject areas, performance on Science was highest on average (followed closely by Writing). Alternatively, mobile students tend to be more antisocial, shy or withdrawn, and have lower classroom participation (Gruman et al., 2008), which could put them at a greater disadvantage in Science classes which often incorporate laboratory and group-based work.

The estimated effects of student mobility presented here have important implications for AYP as defined in NCLB, in that mobility is associated with lower rates of proficiency. In support of this, a crude estimation of the potential impact of this mobility-related academic performance on AYP was performed. Re-calculations of AYP after excluding all mobile students increased the proficiency rate in the CAPT Reading section from 70.5 to 76.2% (*AYP* = 80%), and increased the proficiency rate in the Math section from 66.1 to 71.7% (*AYP* = 80%). While mobility is only one of potentially many factors impacting this school's AYP, the current findings indicate that, though lower rates of proficiency on parts of the CAPT, mobility is likely to impact AYP figures to some extent. Thus, AYP goals are likely to create some additional burden for schools that have high rates of student mobility; policymakers should consider this factor in their work on accountability measures for schools.

### Strengths

The current findings are strengthened by the use of administrative, rather than survey data. A notable advantage of administrative data from the school district is the high retention of the sample. Though data were not available on students that moved *away from* the school district, the inclusion of students moving *into* the school district is particularly beneficial since mobile students have a stronger tendency to drop out of studies. Thus, administrative data provides a rare opportunity for studying the population of mobile students, who are both at risk for low academic performance and for dropping out of survey studies. Further, administrative data provides accurate data rather than relying on self-report (e.g., of standardized test performance).

Additionally, the use of propensity score methods allows a comparatively more accurate isolation of the effect of mobility on CAPT performance, and in that way offers an advantage over many previous studies. While there are important shortcomings in the dataset that preclude concluding a true, unbiased causal effect of mobility (see Section Limitations below), we argue that the use of propensity score methods allows a less-biased estimate of the effect of mobility, relative to conventional approaches of merely controlling for confounding variables, which can lead to severely biased results when they are unevenly distributed across comparison groups. Further, two different propensity score methods for causal inference were used, which show the robustness of the detrimental effect of student mobility on Writing performance.

Further, the particular school used in the current study faces very high levels of student mobility, due to the redistricting imposed on Connecticut public schools by *Sheff v. O'Neill*. This allows for the opportunity to more accurately assess the effects of mobility.

Finally, although the sample contained a majority (55%) of White students, it is much more diverse than Connecticut on average (79% White), with African Americans being especially well-represented (29% in sample vs. 10% in Connecticut; Census Bureau, 1999)^1^.

### Limitations

The present findings should be considered within the context of study limitations. First, the current measure of mobility was constrained by available data in several ways. Specifically, complete data on students in the school district was available beginning in 2006 when the cohort of interest (i.e., those taking the CAPT exam in 2010) were in the 6th grade. This limitation may have reduced the estimated causal effects of mobility, given that any student transferring into the district in grade 6 or earlier would be classified as “stable.” Additionally, data on academic performance of mobile students prior to entering this school district are not available, which is an important confounding variable of mobility's effect on grade 10 standardized test performance. However, the use of socioeconomic indicators (i.e., free/reduced lunch eligibility), which correlates with academic performance, is likely to at least partially account for this unmeasured confounding.

A second important data limitation is the small number of potential confounding variables that are available. A critical assumption of propensity score methods is that there are no unmeasured confounding variables, and it is highly possible that this assumption is violated in these analyses since the only available variables were demographic and background characteristics. The current analyses have mitigated this to the extent possible by also matching on interactions between some of these variables, but it is still likely that other confounding variables exist (e.g., prior academic performance, individual learning styles, and teacher effects). However, though the current findings may contain residual bias, this study still has an advantage over many other studies which are most likely more biased due to their use of conventional approaches (i.e., statistically controlling for confounding variables, despite large differences in distribution across comparison groups). Another consideration is the trade-off between administrative data and survey data: though administrative data typically has a limited number of variables, its major benefit is that it contains complete student information that is not limited by challenges of individual consent or follow-up. Collecting data on additional confounding variables would reduce bias, but would also drastically affect the cost and participation in the study, particularly of the high-risk (i.e., mobile) students. Taken together, the current findings represent a case study using administrative data, and thus provide an important counterpoint to many survey-based studies on student mobility. Finally, the current study demonstrates the importance of future research to evaluate additional variables, such as what role the quality of the student's previous school, reasons for the school transfer, total number of school moves, and additional family factors might play.

A third major limitation is that this case study has limited generalizability to other students and school districts. The current data were drawn from an individual school district representing a generally middle class catchment area with a median household income of $47,162, only slightly above the National median of $46,236 and below the median household income for the State of Connecticut, of $53,935. It is possible that the causal effect of mobility may be different for other higher- or lower-income communities. For example, previous research has found that the circumstances surrounding a school change are different in low vs. high income communities and that these circumstances moderate the impact of the transfer on achievement (Adam, 2004). Within school systems supporting higher socio-economic communities, families are more likely to move into the district in pursuit of better educational or employment opportunities; however, within school systems supporting the lowest income communities, mobility is more likely to result from familial, or financial instability (Adam, 2004). It is possible that mobility more severely impacts low-income students (Temple and Reynolds, 1999), which may indicate that the current results using a mainly middle-class sample may *underestimate* the effects of mobility for lower-income populations. Additionally, the impact of *Sheff v. O'Neill* is particular to public schools in Connecticut, and thus mobile students may have different characteristics and reasons for mobility relative to other schools. Together, these characteristics and considerations of the current sample underscore that this study is a very specific case study with limited implications.

### Implications

The present results suggest that large numbers of students transferring into a school district may adversely impact performance on standardized tests, and this in turn has important implications on the school's achievement of AYP goals as originally defined in NCLB. Even in the current study, in which mobility did not seem to correlate with lower academic performance among low-income students, significant mobility of low-income students nevertheless presents a serious challenge for the school, considering that low-income, mobile students tend to have lower academic performance to begin with (Temple and Reynolds, 1999). More generally, school districts have less time with mobile students to assist them in achieving proficiency and making needed adjustments that may better target individual student needs. In addition, students who do not meet proficiency may often require disproportionate resources to assist them in reaching proficiency, which could in turn adversely impact the scores of stable students, a theory that is supported by research showing that in schools with large numbers of mobile students, stable students have lower test scores and that the curriculum moves at a slower pace (Smith et al., 1998; Rumberger, 2003; Gibbons and Telhaj, 2011).

Because NCLB aimed to punish failing schools by withholding funds, its accountability mandate had the potential to exacerbate the challenges faced by struggling schools: high levels of student mobility lower the percent of proficient students in a school district, in turn preventing the achievement of AYP, and ultimately result in funding cuts that will likely make it more difficult for the school to achieve AYP moving forward. This could be further compounded by the parental options tenant of NCLB (Rumberger, 2002), which allows parents to pull their children from failing schools, thereby increasing the proportion of mobile students being served across systems. Thus, the accountability standards imposed by NCLB are likely to disproportionately and negatively affect struggling schools (Brown and Clift, 2010), creating a vicious cycle of increased student mobility, lower AYP, and reduced funding.

The detrimental effects of student mobility may produce serious and unintended consequences of the accountability mandates of NCLB. Though accountability was intended to motivate better performance in schools, student mobility's negative impact on academic performance has the potential to strongly undermine that goal, particularly when the vicious cycle above emerges. As states re-examine these accountability requirements and consequences under the new ESSA, it is essential to fully understand the intended and unintended effects of these mandates in order to alleviate negative consequences under ESSA. As policymakers plan and implement state-specific accountability mandates, they should re-consider AYP requirements in their original form, particularly the penalty of withholding funds, due to the increased burden it places on struggling students and schools.

## Ethics statement

This study was exempted from ethical approval by the Wesleyan University IRB.

## Author contributions

AS contributed to study design, conducted data analysis and interpretation, drafted the manuscript, and revised the manuscript. EE contributed to study design, conducted data analysis, and drafted the manuscript. LD contributed to study design and revising the manuscript. ES contributed to data analysis and revising the manuscript. JS contributed to study design and revising the manuscript. DC contributed to data analysis, interpretation, and revising the manuscript. MO contributed to study design, data acquisition, and revising the manuscript. All authors approved the final version and agree to be accountable for their work.

## Funding

This research was supported by grant 0942246 from the National Science Foundation, Transforming Undergraduate Education in Science, Technology, Engineering and Mathematics (TUES) and the Lauren B. Dachs Grant in Support of Interdisciplinary Research in the Social Impacts of Science.

### Conflict of interest statement

The authors declare that the research was conducted in the absence of any commercial or financial relationships that could be construed as a potential conflict of interest.

## Footnotes

^1^Census Bureau. Available online at: http://www.census.gov/

## References

[B1] Adam E. K. (2004). Beyond quality: parental and residential stability and children's adjustment. Curr. Dir. Psychol. Sci. 13, 210–213. 10.1111/j.0963-7214.2004.00310.x

[B2] Alexander K. L. Entwisle D. R. Dauber S. L. (1996). Children in motion: school transfers and elementary school performance. J. Educ. Res. 90, 3–12. 10.1080/00220671.1996.9944438

[B3] Alliance for Excellent Education (2006). Reading and Writing in the Academic Content Areas. Washington, DC.

[B4] Applebee A. N. Langer J. A. (2009). EJ Extra: what is happening in the teaching of writing? English J. 98, 18–28.

[B5] Brown A. B. Clift J. W. (2010). The unequal effect of adequate yearly progress evidence from school visits. Am. Educ. Res. J. 47, 774–798. 10.3102/0002831210374644

[B6] Coleman J. S. (1988). Social capital in the creation of human capital. Am. J. Sociol. 94, S95–S120. 10.1086/228943

[B7] Connecticut vs. Spellings (2005). United States District Court, Connecticut.

[B8] Dauber S. L. Alexander K. L. Entwisle D. R. (1996). Tracking and transitions through the middle grades: channeling educational trajectories. Sociol. Educ. 69, 290–307. 10.2307/2112716

[B9] Dee T. S. Jacob B. (2011). The impact of No Child Left Behind on student achievement. J. Policy Anal. Manage. 30, 418–446. 10.1002/pam.20586

[B10] Desimone L. M. (2013). Teacher and administrator responses to standards-based reform. Teach. Coll. Rec. 115, 1–53.

[B11] Downing S. M. (2002). Threats to the validity of locally developed multiple-choice tests in medical education: construct-irrelevant variance and construct underrepresentation. Adv. Health Sci. Educ. 7, 235–241. 10.1023/A:1021112514626 12510145

[B12] Ellickson P. L. McGuigan K. A. (2000). Early predictors of adolescent violence. Am. J. Public Health. 90:566. 10.2105/AJPH.90.4.566 10754971 PMC1446195

[B13] Engec N. (2006). Relationship between mobility and student performance and behavior. J. Educ. Res. 99, 167–178. 10.3200/JOER.99.3.167-178

[B14] Gasper J. DeLuca S. Estacion A. (2012). Switching schools: revisiting the relationship between school mobility and high school dropout. Am. Educ. Res. J. 49, 487–519. 10.3102/0002831211415250 25554706 PMC4279956

[B15] Gibbons S. Telhaj S. (2011). Pupil mobility and school disruption. J. Public Econ. 95, 1156–1167. 10.1016/j.jpubeco.2011.03.004

[B16] Grigg J. (2012). School enrollment changes and student achievement growth: a case study in educational disruption and continuity. Sociol. Educ. 85, 388–404. 10.1177/0038040712441374

[B17] Gruman D. H. Harachi T. W. Abbott R. D. Catalano R. F. Fleming C. B. (2008). Longitudinal effects of student mobility on three dimensions of elementary school engagement. Child Dev. 79, 1833–1852. 10.1111/j.1467-8624.2008.01229.x 19037953 PMC3870003

[B18] Hansen B. B. (2004). Full matching in an observational study of coaching for the SAT. J. Am. Stat. Assoc. 99, 609–618. 10.1198/016214504000000647

[B19] Herbers J. E. Cutuli J. J. Supkoff L. M. Heistad D. Chan C. K. Hinz E. (2012). Early reading skills and academic achievement trajectories of students facing poverty, homelessness, and high residential mobility. Educ. Res. 41, 366–374. 10.3102/0013189X12445320

[B20] Ho D. E. Imai K. King G. Stuart E. A. (2011). MatchIt: nonparametric preprocessing for parametric causal inference. J. Stat. Softw. 42, 1–28. 10.18637/jss.v042.i08

[B21] Jennings J. L. Bearak J. M. (2014). “Teaching to the Test” in the NCLB era: how test predictability affects our understanding of student performance. Educ. Res. 43, 381–389. 10.3102/0013189X14554449

[B22] Jensen J. McDaniel M. Woodard S. Kummer T. (2014). Teaching to the test…or testing to teach: exams requiring higher order thinking skills encourage greater conceptual understanding. Educ. Psychol. Rev. 26, 307–329. 10.1007/s10648-013-9248-9

[B23] Jones G. M. Jones B. D. Hargrove T. (2003). The Unintended Consequences of High-Stakes Testing. Lanham, MD: Rowman and Littlefield Publishers.

[B24] Kerbow D. Azcoitia C. Buell B. (2003). Student mobility and local school improvement in Chicago. J. Negro Educ. 72, 158–164. 10.2307/3211299

[B25] Kim J. S. Sunderman G. L. (2005). Measuring academic proficiency under the no child left behind act: implications for educational equity. Educ. Res. 34, 3–13. 10.3102/0013189X034008003

[B26] Liu L. Neilson W. S. (2011). High scores but low skills. Econ. Educ. Rev. 30, 507–516. 10.1016/j.econedurev.2010.12.004

[B27] MacPherson S. (2009). Teaching biology or teaching to the test?: how high stakes standardized tests impact the biology classroom. Int. J. Learn. 16, 525–534. 26753047

[B28] Mehana M. Reynolds A. J. (2004). School mobility and achievement: a meta-analysis. Child. Youth Serv. Rev. 26, 93–119. 10.1016/j.childyouth.2003.11.004

[B29] Nelson P. S. Simoni J. M. Adelman H. S. (1996). Mobility and school functioning in the early grades. J. Educ. Res. 89, 365–369. 10.1080/00220671.1996.9941340

[B30] Parke C. S. Kanyongo G. Y. (2012). Student attendance, mobility, and mathematics achievement in an urban school district. J. Educ. Res. 105, 161–175. 10.1080/00220671.2010.547231

[B31] Pribesh S. Downey D. B. (1999). Why are residential and school moves associated with poor school performance? Demography 36, 521–534. 10.2307/2648088 10604079

[B32] Rosenbaum P. R. (2002). Observational Studies, 2nd Edn. New York, NY: Springer Verlag.

[B33] Rosenbaum P. R. (2010). Design of Observational Studies. New York, NY: Springer Science+Business Media, LLC.

[B34] Rumberger R. W. (2002). Student Mobility and Academic Achievement. Champaign, IL: ERIC Clearinghouse on Elementary and Secondary Education.

[B35] Rumberger R. W. (2003). The causes and consequences of student mobility. J. Negro Educ. 72, 6–21. 10.2307/3211287

[B36] Rumberger R. W. Larson K. A. (1998). Student mobility and the increased risk of high school dropout. Am. J. Educ. 107, 1–35. 10.1086/444201

[B37] Ryan J. E. (2004). Perverse incentives of the no child left behind act. NYUL Rev. 79:932 10.2139/ssrn.476463

[B38] Sekhon J. S. (2011). Multivariate and propensity score matching software with automated balance optimization: the matching package for R. J. Stat. Softw. 42, 1–52. 10.18637/jss.v042.i07

[B39] Simpson G. A. Fowler M. G. (1994). Geographic mobility and children's emotional/behavioral adjustment and school functioning. Pediatrics 93, 303–309. 8121745

[B40] Smith J. B. Smith B. Bryk A. S. (1998). Setting the Pace: Opportunities to Learn in Chicago Public Elementary Schools. Chicago, IL: Consortium on Chicago School Research, University of Chicago.

[B41] South S. J. Haynie D. L. Bose S. (2007). Student mobility and school dropout. Soc. Sci. Res. 36, 68–94. 10.1016/j.ssresearch.2005.10.001

[B42] Strand S. Demie F. (2006). Pupil mobility, attainment and progress in primary school. Br. Educ. Res. J. 32, 551–568. 10.1080/01411920600775191

[B43] Strand S. Demie F. (2007). Pupil mobility, attainment and progress in secondary school. Educ. Stud. 33, 313–331. 10.1080/03055690701423184

[B44] Temple J. A. Reynolds A. J. (1999). School mobility and achievement: longitudinal findings from an urban cohort. J. Sch. Psychol. 37, 355–377. 10.1016/S0022-4405(99)00026-6

[B45] The National Commission on Writing (2002). The Neglected “R”: The Need for a Writing Revolution. New York, NY.

[B46] Thompson S. M. Meyers J. Oshima T. C. (2011). Student mobility and its implications for schools' adequate yearly progress. J. Negro Educ. 80, 12–21.

[B47] U.S. Department of Agriculture (2011). Income Eligibility Guidelines. Food and Nutrition Service Home Page. Available online at: http://www.fns.usda.gov/cnd/governance/notices/iegs/iegs.htm

[B48] U.S. Department of Education (2012). Student Mobility and Academic Achievement. in *ESEA Flexibility Request: Connecticut*. Washington, DC: ERIC Clearinghouse on Elementary and Secondary Education.

[B49] Voight A. Shinn M. Nation M. (2012). The longitudinal effects of residential mobility on the academic achievement of urban elementary and middle school students. Educ. Res. 41, 385–392. 10.3102/0013189X12442239

[B50] Wright D. (1999). Student mobility: a negligible and confounded influence on student achievement. J. Educ. Res. 92, 347–353. 10.1080/00220679909597618

[B51] Zhang H. C. Cowen D. J. (2009). Mapping academic achievement and public school choice under the No Child Left Behind legislation. Southeast. Geogr. 49, 24–40. 10.1353/sgo.0.0036

